# Parental identity formation in mothers is linked to borderline and depressive symptoms: A person-centered analyses

**DOI:** 10.3389/fpsyg.2023.1086947

**Published:** 2023-01-26

**Authors:** Konrad Piotrowski

**Affiliations:** Center for Research on Personality Development, SWPS University, Poznań, Poland

**Keywords:** parental identity, depressive symptoms, borderline symptoms, U-MICS, person-centered

## Abstract

The formation of a stable parental identity is an important developmental task which parents face. Difficulties in this process can significantly decrease the quality of life and hinder the fulfillment of the parental role. The present study analyzed whether parental identity status, based on the three identity processes from the Meeus-Crocetti model, is related to the severity of borderline and depressive symptoms. Four hundred and fifty-nine mothers aged 18–40 (*M* = 32.41, *SD* = 5.09) participated in the cross-sectional study. The results of the cluster analysis revealed the existence of five different parental identity statuses: Achievement, Foreclosure, Searching moratorium, Diffussion, and Moratorium. Significant differences were also observed between parents with different statuses in terms of borderline and depressive symptoms. The study confirmed predictions that mental health difficulties among parents co-occur with low identification with parenthood.

## Introduction

Being a parent is a complex and ambiguous experience that may be related both to satisfaction and self-development and to stress, burden, and burnout (Antonucci and Mikus, 1988; Hutteman et al., 2014; Doss and Rhoades, 2017; Mikolajczak et al., 2021). Recently, it was suggested that the sense of parental identity can explain these diverse trajectories in parents (Piotrowski, 2018, 2021a; Meca et al., 2020; Schrooyen et al., 2021). When a parent has too few resources (psychological, social, relational, financial, etc.; Mikolajczak et al., 2018) or is experiencing mental health difficulties (Meca et al., 2020), parenting can become a stressful and unpleasant experience, leading to parental identity disturbances that can manifest itself even in regret about having become a parent (Donath, 2015; Piotrowski, 2021b). If such a state is prolonged it can lead to further decline in mental health (Schrooyen et al., 2021) and to violence against children (East et al., 2012). Since a stable sense of parental identity is one of the foundations of a parent’s quality of life (Fadjukoff et al., 2016; Schrooyen et al., 2021), knowledge of its relationship to mental health is of great practical and scientific importance.

As only a few studies have been conducted to date on the relationship between symptoms of psychopathology and parental identity (Piotrowski, 2018; Meca et al., 2020; Schrooyen et al., 2021), the present study aimed to fill the existing gaps in our knowledge on this topic. In the present study, I examined the link between parental identity and symptoms of borderline personality disorder (sBPD; a subtype of emotionally unstable personality disorder), which has not been studied before in this context, and symptoms of depression, which has only been examined in two studies so far (Meca et al., 2020; Schrooyen et al., 2021). Since sBPD and depressive symptoms are among the most common personality and affective mental health issues in the world, they are certainly a daily experience for tens of millions of parents. Assessing the relationship between these health difficulties and parental identity formation is therefore of great scientific and practical importance.

The present study focused specifically on mothers’ experiences due to the fact that the role of parent occupies a more important position in their self-image (Kerpelman and Schvaneveldt, 1999; Williams and Kelly, 2005), making women more prone to experiencing severe parental stress (Simon, 1992; Hildingsson and Thomas, 2013). The study also found that mothers are more likely to experience parental burnout, although the reasons for these differences still require further research (Roskam et al., 2018; Sorkkila and Aunola, 2020). Since a well-formed parental identity appears to be a protective factor against stress (Schrooyen et al., 2021; Piotrowski, 2022), a good understanding of the determinants of its development in mothers and the links to mental health is necessary. Although studies on the prevalence of borderline personality disorder among men and women are ambiguous (some studies show higher prevalence among women and others show similar prevalence among men and women, Ellison et al., 2018; Dehlbom et al., 2022), for depression, the prevalence among women is twice as high as among men (Hyde et al., 2008; Hyde and Mezulis, 2020), putting mothers at particular risk. The present study aimed to better understand the relationship between mothers’ parental identity and their mental health.

### Processual approach to parental identity

When a person becomes a parent this new role changes their sense of identity by adding a new domain into it. Parental identity is being defined as the degree to which the role of a parent is seen as important when compared to other social roles (Maurer et al., 2001), as a dynamic self-definition as a parent (Delmore-Ko, 2000), or firmness of commitment to the parenting domain (Fadjukoff et al., 2016). Recently, Piotrowski (2018, 2021a) has used the Meeus-Crocetti processual identity model (Crocetti et al., 2008) as a starting point and defined parental identity as the degree of commitment to and identification with the parental role. According to this processual approach, when a person becomes a parent, a process of identity commitment takes place, resulting in a baseline level of self-confidence and identification with this new role. When a parental role is undertaken, two different forms of exploration occur: in-depth exploration (i.e., the extent to which parents think actively about a child and parenting and deepen their knowledge about commitments made) and reconsideration of commitment (i.e., a comparison of being a parent with its alternative, either not having a child or being childless). A high reconsideration of commitment is an indicator of disappointment with a parental role and a parental identity crisis. The dynamics between these three identity processes (commitment, in-depth exploration, and reconsideration) may lead to strengthening of parental identity commitment when it is further endorsed by in-depth exploration (commitment and in-depth exploration are positively correlated; Piotrowski, 2018, 2020) or to parental identity disturbances when a parent experiences a state of doubts, regrets, and even starts to think that childlessness would be a better choice (in such a case high reconsideration and low commitment is observed).

### Parental identity statuses

In his subsequent study Piotrowski (2021a) showed that similarly to other identity domains studied within the Meeus-Crocetti model, such as education, work, and partner/best friend relationship (Crocetti et al., 2008), in the parental domain also the five different identity statuses (specific configuration of the three identity processes in a person) can be distinguished: *Achievement* (high commitment and in-depth exploration, low reconsideration of commitment), *Foreclosure* (high commitment, low exploration and reconsideration of commitment), *Searching moratorium* (low commitment and reconsideration of commitment, and high in-depth exploration), *Difussion* (low commitment, in-depth exploration, and reconsideration of commitment), and *Moratorium* (low commitment and in-depth exploration, high reconsideration of commitment). The study showed that while women were more likely than men to develop the most adaptive Achieved identity status, at the same time about a quarter of mothers were characterized by relatively low identity commitment (Diffusion and Moratorium statuses). Piotrowski (2021a) study showed that there are significant differences between parents with different identity statuses in terms of personality traits and quality of life. Parents with Achievement and Foreclosure statuses are characterized by high agreeableness and conscientiousness and low neuroticism (Costa and McCrae, 1992), high optimism and satisfaction with life. Different results were observed for parents with Moratorium status. In Piotrowski (2021a) study, they were characterized by high indecisiveness, pessimism, perfectionism and neuroticism. Parents with Searching moratorium and Diffusion statuses, on the other hand, in terms of adjustment and quality of life were between these extreme groups. The difficulties in the functioning of parents with Moratorium status observed by Piotrowski (2021a) indeed suggest that the severity of psychopathology symptoms among them may be high. The current study is the first to verify this empirically.

### Parental identity processes and psychopathology

Formation of a stable sense of identity is one of the key developmental tasks facing a person (Erikson, 1950). At the same time, identity formation is a task that requires considerable resources. In order to achieve a stable sense of identity, it is necessary to make many decisions, to be able to cope with difficulties, to be faithful to the choices made (Erikson, 1950). Unsurprisingly, people struggling with mental health issues are found to have greater difficulties in this regard (Klimstra and Denissen, 2017). Although few studies have been devoted to cause-and-effect relationships so far, it can be predicted that the relationship between identity development and psychopathology is bidirectional. Crocetti et al. (2013) observed that higher levels of externalizing symptoms in adolescents predict difficulties with their sense of identity several years later. On the other hand, the course of identity development has also been shown to be a good predictor of depression symptom severity 5 years later (Schwartz et al., 2012). Unfortunately, the vast majority of studies on the relationship between psychopathology and identity have been conducted with participants in adolescence and emerging adulthood, with almost no attention paid to one of the most important identity domains in adulthood, namely parental identity. Only a handful of studies published up to date had addressed this issue.

Schrooyen et al. (2021) studied parents aged 18 to 83 during the COVID-19 pandemic and observed that parents with a better-formed definition of themselves as parents (with a stronger commitment) were characterized by higher life satisfaction and lower levels of depressive and anxiety symptoms. Also, studies based on the three-dimensional model described above that was conducted in Poland (Piotrowski, 2018, 2020, 2021b) and in the US (Meca et al., 2020) suggest that low commitment and high reconsideration of commitment in the parental domain are associated with high anxiety (Piotrowski, 2018; Meca et al., 2020), narcissism (Piotrowski, 2021b), perfectionism (Piotrowski, 2020), and depressive symptoms (Meca et al., 2020).

Among its different correlates, depressive and anxiety symptoms are also closely related to personality pathology such as borderline personality disorder (BPD; Gunderson and Philips, 1991; Koenigsberg et al., 1999), which has not yet been studied in the context of parental identity. People with BPD, experience highly unstable emotional and relational life, are characterized by strong and unpredictable reactions, risky behaviors, unstable self-esteem and also a fragile sense of identity (Gunderson and Philips, 1991; Bogaerts et al., 2021). Thus, it can be assumed that parents with high borderline symptoms may also have considerable difficulty forming a stable identity of themselves as parents. The present study is the first to verify this hypothesis within the processual approach to identity development.

As all studies to date on the relationship between parental identity and symptoms of psychopathology have been conducted using a variable-centered approach (Piotrowski, 2018, 2020, 2021b; Meca et al., 2020; Schrooyen et al., 2021), the current study used a person-centered approach. This second approach provides a better understanding of how the interaction of specific identity processes – known as identity status (Marcia, 1966) – relates to an individual’s functioning. This allowed us to specify what differences in terms of parental identity development exist between parents and what type (status) of parental identity is most strongly associated with psychopathology symptoms.

### Research problem and hypotheses

The conceptualization of parental identity used in this study was based on the three-dimensional processual approach (Crocetti et al., 2008; Piotrowski, 2018, 2020). I analyzed whether mothers with different parental identity status (i.e., configuration of the three identity processes; Crocetti et al., 2008) differ in BPD and depressive symptoms. Up to date, only in one study (Piotrowski, 2021a) parental identity processes have been analyzed with the use of the person-centered approach, however neither BPD nor depressive symptoms were studied in this context.

It was hypothesized that, as in an earlier study (Piotrowski, 2021a), individual differences in parental identity development would be best explained by five different identity statuses (described above), and that mothers with different statuses will differ in the level of BPD and depressive symptoms. I assumed that Achievement and Foreclosure statuses will be associated with the lowest level of BPD and depressive symptoms, while the Moratorium status will be associated with the highest BPD and depressive symptoms. Mothers in Searching moratorium and Diffusion will be placed between these extreme groups. Such a result would be consistent with previous observations (Piotrowski, 2021a), and would suggest that the differences between parental identity statuses with respect to psychopathology are similar to those with respect to personality traits.

## Methods

### Participants

A total of 459 Polish mothers aged 18–40 (*M*_age_ = 32.41, *SD* = 5.09) took part in the study. Participants were well-educated (69.3% had higher education diploma), their financial situation was also rather good (38.6% stated that they did not have any financial problems; 56.4% stated that only sometimes they had financial difficulties, and only 5% of the participants described their situation as not good); 71.2% were married, and 21.1% were in an informal relationship, 7.6% were single. Participants had from one to four children, aged from 1 month to 21 years. However, in the most cases the studied women had rather young children: 81.6% of them had the oldest child not older than 10, and 96.5% of the participants had children younger than 15; the mean age of the children was 4.92, *SD* = 3.87. Participants were recruited through social media advertisements (about half of the mothers) and through a nationwide research panel.

### Measures

#### Parental identity

The three identity processes postulated in the Meeus-Crocetti model (Crocetti et al., 2008) were measured with the Utrecht-Management of Identity Commitments Scale-Parental Identity (U-MICS-PI; Piotrowski, 2018, 2020). The questionnaire consists of 13 items, five for Commitment (e.g., Being a parent allows me to face the future with optimism), five for In-depth exploration (e.g., I make a lot of effort to keep finding out new things about my child/children), and three items for Reconsideration of commitment (e.g., I often think that not having a child/children would have made my life more interesting). Items are rated on a 5-point Likert scale. Cronbach’s Alpha and McDonald’s Omega of the subscales were, respectively, 0.91 and 0.91 for commitment, 0.70 and 0.68 for exploration and 0.92 and 0.93 for reconsideration of commitment. Also, the composite reliability of each of the subscales exceeded 0.70. The slightly lower consistency of the in-depth exploration subscale is related to the scale design and is due to the wide variety of behaviors the items address. Confirmatory factor analysis performed with AMOS 28 confirmed the three-factor structure of the questionnaire: CFI = 0.969, RMSEA = 0.063, SRMR = 0.045 (Hu and Bentler, 1999).

#### Borderline personality symptoms

BPD symptoms were measured with the Polish adaptation of the Borderline Personality Disorder Checklist (BPD Checklist; Bloo et al., 2017), a 47-item questionnaire that assesses an intensity of BPD symptoms (e.g., Feeling bored or empty inside) during the previous month. The items that comprise the questionnaire are based on the conceptualization of borderline personality as depicted in the DSM-IV classification. Items are rated on a 5-point Likert scale (from *not at all* to *extremely*); the sum of all items is an overall index of the subjective burden caused by BPD symptoms. Cronbach’s alpha and McDonald’s Omega of the overall score were 0.94 and 0.95, respectively, and composite reliability was 0.77, indicating that the overall scale was highly consistent.

#### Depressive symptoms

Depressive symptoms were assessed with the Center for Epidemiological Studies – Depression scale (CES-D; Radloff, 1977; Jankowski, 2016). The scale consists of 20 items reflecting a frequency of symptom occurrence during the past week (from 0-*rarely or none of the time* to 3-*most or all of the time*). The items cover various aspects of depression, i.e., somatic symptoms, depressed affect, positive affect, and interpersonal relations (e.g., I did not feel like eating; my appetite was poor). The sum of all items is used as an indicator of depression symptomatology. In the present study, Cronbach’s Alpha, McDonald’s Omega, and composite reliability of the scale were 0.93,0.94, and 0.76, respectively, indicating that the overall scale was highly consistent.

### Procedure

The study was conducted online with a convenience sample recruited using a snowball method. All participants signed the informed consent. The project was approved by the Departmental Ethics Committee at the author’s institution. The data was collected in the first half of 2019, before the start of the COVID-19 pandemic.

### Statistical analysis

At the beginning, missing data analysis was performed. It turned out that missing values occurred only occasionally and were missing completely at random (Little’s MCAR test was insignificant). They were replaced with the mean value for the subscale. An analysis of the distribution of the variables revealed that all the variables studied had a distribution close to normal (skewness and kurtosis ranging from −2 to 2). In the first step, a correlation analysis was conducted between the variables under study. In the second step, the parental identity statuses were distinguished (three identity variables were used in this procedure: commitment, in-depth exploration, and reconsideration of commitment) with the use of the two-step cluster analysis suggested by Gore (2000) which is often used in identity studies (e.g., Crocetti et al., 2008; Bogaerts et al., 2021). Since the presence of collinearity between the variables included in the cluster analysis could negatively affect the results, this issue was first investigated. It turned out, however, that collinearity between the three identity variables did not occur (the VIF value in each case was close to 1), allowing the cluster analysis to proceed. In the first step, the results on the three dimensions of parental identity were standardized, and outliers (17 persons) were removed (univariate outliers were individuals with + − 3*SD* on any dimension, and multivariate outliers were individuals with high Mahalanobis distance, *p* < 0.001). Subsequently, a hierarchical analysis with the use of Ward’s method with squared Euclidean distances (Gore, 2000) was conducted. In subsequent analyses, 3, 4, 5 and 6 clusters were distinguished. The initial centers of clusters obtained in the hierarchical analysis were used in the next step as non-random starting points in an iterative k-means cluster analysis (Gore, 2000). The clusters distinguished in such a way were subjected to the evaluation in order to select the most optimal solution (i.e., optimal number of clusters). The most optimal solution was chosen basing on their theoretical validity, as it was assumed that the solutions obtained should be close to the results of other studies (Crocetti et al., 2008; Piotrowski, 2021a), parsimony (each cluster should be characterized by a different configuration of the dimensions), and explanatory power (the percentage of the variance of each identity dimension explained by the given cluster solution should be at least 50%). Then, a multivariate analysis of variance (MANOVA) was conducted in order to compare the clusters in respect of BPD and depressive symptoms.

## Results

### Descriptive statistics and correlational analysis

Descriptive statistics and correlational analysis are presented in Table 1. Correlations between the identity processes were similar as observed in the previous studies (Crocetti et al., 2008; Piotrowski, 2018). Parental identity commitment was negatively, moderately related to BPD and depressive symptoms; in-depth exploration was not related to mental health; and reconsideration of commitment correlated positively, moderately both with BPD and depressive symptoms. A strong, positive relationship between BPD and depressive symptoms in mothers was also observed.

**Table 1:** Pearson’s *r* correlation between the analyzed variables (*N* = 459).

	*M*	*SD*	Min	Max	1	2	3	4	5
1. Commitment	3.32	0.95	1.00	5.00	-	0.30***	−0.58***	−0.39***	−0.44***
2. In-depth exploration	4.08	0.55	2.00	5.00		-	−0.17***	0.01	−0.06
3. Reconsideration of commitment	1.78	0.98	1.00	5.00			-	0.43***	0.40***
4. Borderline symptoms	78.52	21.67	49	178				-	0.76***
5. Depressive symptoms	16.95	12.70	0.00	56					-

*p < 0.05, **p < 0.01, ***p < 0.001.

### Parental identity statuses and mental health

**Figure 1:**
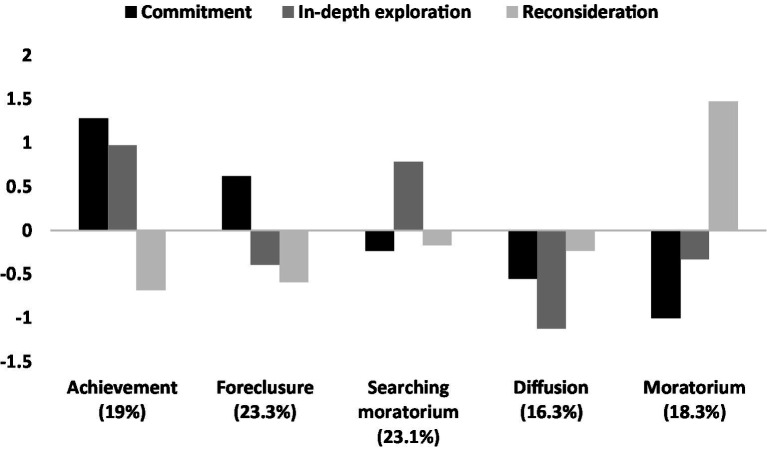
Parental identity statuses in the study sample.

Comparing of the different cluster solutions (three, four, five, and six clusters), similarly to the Piotrowski (2021a) study, led to the conlusion that five clusters of parental identity are the most optimal. When I go from three to five clusters, every step resulted in a new configuration; however in the six-clusters solution two clusters were highly similar to each other, which suggested that the five clusters described the differences between the parents well enough. Although four clusters explained about 60% of the variance of each identity dimension, meeting the criterion of explained variance, the five-cluster solution not only yielded a new and specific group, but also all clusters were similar to those described in earlier studies meeting the criterion of theoretical validity (Crocetti et al., 2008; Piotrowski, 2021a,b). The five cluster solution, presented in Figure 1, explained from 65 to 72% of the variance of the particular dimensions. The obtained identity statuses were comparable to those observed earlier by Piotrowski in the same identity domain (Piotrowski, 2021a), and by Crocetti et al. (2008) in the educational and relational identity domains.

Achievement and Foreclosure, the two most adaptive identity statuses (Crocetti et al., 2008) characterized by high commitment and low reconsideration, together constituted 42.3% of the sample; on the other end, Moratorium (low commitment, moderate exploration, high reconsideration) status characterized 18.3% of the mothers. The least frequent was the status of Diffusion (16.3%).

Multivariate analysis of variance (MANOVA) revealed a significant multivariate effect of the identity status on BPD and depressive symptoms, Wilks’ λ = 0.80, *F*(8, 872) = 12.72, *p* < 0.001, *η*^2^ = 0.11. In Table 2 the results of univariate analyses are presented. As hypothesized, Achievement and Foreclosure in the parental domain were associated with the lowest results on BPD and depressive symptoms. Similarly to the results of Piotrowski (2021a), these two clusters do not differed from each other. In line with the assumptions, Moratorium status defined by low commitment, average exploration, and high reconsideration of commitment was associated with higher BPD and depressive symptoms then in the other clusters (except for insignificant difference between Moratorium and Diffusion on depressive symptoms). Table A1 presents descriptive statistics of the variables separately for each identity status.

**Table 2:** Differences between parental identity statuses on BPD and depressive symptoms.

	Parental identity statuses					*F*	Eta^2^
	Achievement (*n* = 84, 19%)	Foreclosure (*n* = 103, 23.3%)	Searching moratorium (*n* = 102, 23.1%)	Diffusion (*n* = 72, 16.3%)	Moratorium (*n* = 81, 18.3%)		
BPD symptoms	71.24^a^ (19.65)	68.57^a^ (15.42)	80.88^b^ (22.07)	79.88^b^ (20.14)	91.41^c^ (21.72)	18.19***	0.14
Depression	11.01^a^ (9.76)	11.39^a^ (9.70)	17.63^b^ (13.04)	21.06^b,c^ (11.90)	24.23^c^ (13.10)	21.79***	0.17

Mean values with different indices are significantly different at least at the *p* < 0.05 level (*post-hoc* test: Tukey HSD); SD values are given in parentheses. Because Levene’s test revealed a violation of the assumption of equality of variance between the compared groups, the same analysis was repeated using the non-parametric Kruskall–Wallis test with the Bonferroni correction for multiple testing. The results proved to be identical to the MANOVA results shown in the table.

## Discussion

It has been previously suggested that psychopathology is in close, reciprocal relationships with identity (e.g., Wiley and Berman, 2013), however this idea is still understudied from the developmental and processual perspectives. In the present research I focused on a better understanding of the association between one of the least known identity domains (Piotrowski, 2018), i.e., parental identity and its links with symptoms of such mental health difficulties as borderline personality and depression.

Although studies suggest that women are slightly more likely than men to form an Achieved identity status that is highly adaptive, at the same time, up to 25% of mothers are characterized by relatively low identity commitment in the parental domain (Piotrowski, 2021a), and about 10–13% of them regret having decided to have children (Piotrowski, 2021b). The obtained results support the hypothesis that the development of a stable sense of parental identity, as manifested in a strong identification with the role of parent, may be hindered when the mother is struggling with mental health issues such as borderline personality or depression. An earlier study by Meca et al. (2020) suggests that the situation may be similar among fathers, but further research is needed to confirm this.

Consistently with studies focused on other identity domains (Bogaerts et al., 2021), mothers with higher borderline and depressive symptoms turned out to be at higher risk for lower commitment and identification with a parental role, stronger doubts about being a parent, and dreaming about the ‘life without kids’ (Piotrowski, 2021b). The present study, like an earlier study by Meca et al. (2020) on a sample of U.S. parents, also confirmed that the dimension of exploration is not closely related to parents’ mental health. While Meca et al. (2020) observed a small positive association with anxiety levels, no such association was observed for symptoms of depression. Thus, it is possible that the tendency to explore parental identity issues is determined by more general personality traits rather than mental health problems. For example, Piotrowski (2021a) showed that parents with a high tendency to explore parenthood issues are characterized by higher extraversion and agreeableness. It is advisable for future studies of parental identity development to deepen our understanding of the relationship of exploration with psychopathology.

As identity processes are related to each other, person-centered approach was used in the present study in order to distinguish different subgroups/types, i.e., identity statuses (Marcia, 1966). The obtained results support Piotrowski (2021a) conclusions that five different statuses of parental identity can be observed, similarly as in other domains (Crocetti et al., 2008). Obtaining the same identity statuses in the mothers’ group as in the different-gender sample (Piotrowski, 2021a), confirms the reliability of the analytical approach used. Mothers with Achievement and Foreclosure statuses, both characterized by high commitment and low reconsideration of commitment, experienced the lowest level of mental health problems, while the highest BPD and depression symptoms were observed in Moratorium status, which is in line with the previous studies (Piotrowski, 2021a).

Particular identity statuses are sometimes seen as different positions on the Eriksonian dimension of identity synthesis-identity confusion (Erikson, 1950; Marcia, 1966). From this perspective, parents in Moratorium status seem to be close to the identity confusion pole, which may be both an effect and a source of their mental health problems. As Piotrowski (2021a) showed parents in Moratorium status are characterized by the highest narcissism, perfectionism, and stressful and conflictual relationships. The present study adds to this picture also the highest borderline and depressive symptoms. Parents in Moratorium status constitute about 20% of the samples that have already been studied within the Meeus-Crocetti model in parental domain (Piotrowski, 2021a), which could translate to a significant group of parents worldwide struggling with parental identity formation. Further cross-cultural studies on this topic and a better understanding of their development is highly recommended.

### Limitations and further research

The present research offers new insights into an important and rarely studied link between mental health in mothers and their parental identity; nevertheless, the results must be assessed in context of several important limitations. First of all, only mothers took part in this study, which makes it difficult to generalize results. Although Meca et al. (2020) did no observe gender differences in the direction of relationships between parental identity, depression, and anxiety, there is still a need to study this topic with men in the sample. Additionally, the study sample was not randomly gathered, which warrants caution when generalizing the results. Secondly, the conclusions are based on cross-sectional data and need to be verified longitudinally. Thirdly, depressive and borderline symptoms were assessed with self-report questionnaires only, which might affect the results. In addition, the questionnaire used to measure borderline symptoms was based on the DSM-IV criteria, and not the DSM-V in which some changes were made to the diagnostic criteria (Samuel et al., 2012). More objective data on mental health should be used in the future. Additionally, the severity of depressive symptoms and BPD were strongly positively correlated. It would be worthwhile for future studies to focus on other forms of mental health problems to increase the generalizability of the results. Fourthly, the in-depth exploration subscale of the U-MICS-PI questionnaire had rather moderate reliability, which could also have affected the results. Fifthly, the lack of a significant relationship between exploration and mental health suggests that mental health difficulties are primarily associated with the level of commitment, that is, strong commitment and low reconsideration of commitment are associated with better mental health, and low commitment and high reconsideration of commitment with worse mental health. As a result, the study did not find much variation between statuses, although they were consistent with predictions and previous studies (Piotrowski, 2021a,b). Future studies using person-centered approaches should shed more light on the differences between Achievement and Foreclosure and Searching moratorium and Diffusion statuses. Sixthly, the present study focused on a symptoms-based approach rather than a diagnostic approach, and therefore the relevance to clinical knowledge is limited. Study participants were not assessed for depression and borderline personality disorder, only for the severity of symptoms that are associated with these disorders. Subsequent studies should focus more on the clinical group and deepen knowledge about the relationship between mental illness and its impact on parental identity development.

## Conclusion

Parenting, in contrary to popular views, is a stressful and requiring activity (Mikolajczak et al., 2018) and such mental health difficulties as high borderline symptoms, or even a borderline personality disorder, and depression can damage social and emotional functioning, and become factors that make every day parental interactions even more difficult. For some parents mental health issues can create a context in which being a parent is not a source of satisfaction and self-development but rather a source of doubts and burden. As a result, the process of parental identity development, that ideally leads a parent to a state of self-confidence, identification with the role, and satisfaction with parenting (Piotrowski, 2018), can also be disrupted and reconsideration of commitment can come to the fore, possibly leading to further deterioration of the parent’s mental health (Schrooyen et al., 2021).

The present study highlights several possible directions for prevention and clinical interventions. Since the period of pregnancy and the first months after the child’s birth are a turning point in parental identity development (Meca et al., 2020), it is advisable to include women experiencing BPD and depression in such forms of support that will lower the risk of the mother regretting her decision to parenthood. The literature provides information on a number of evidence-based early interventions with the potential effect of minimizing the severity of reconsideration of commitment in parental domain, such as infant-parent psychotherapy (Wendland et al., 2014) and mindfulness-based interventions (Bateman and Fonagy, 2010). Since regretting parenthood can initiate a series of subsequent negative changes in a family’s life (East et al., 2012), the goal of early intervention should be to reduce the risk that a parent will begin to view parenthood in this manner. It is also advisable, in clinical work with parents with elevated symptoms of BPD and depression at any stage of their lives, to pay close attention to patients’ attitudes toward parenthood and to detect signals of parental regret (Piotrowski, 2020, 2021b), and to incorporate this into psychotherapeutic work if the issue emerges.

## Data availability statement

The raw data supporting the conclusions of this article will be made available by the authors, without undue reservation.

## Ethics statement

The studies involving human participants were reviewed and approved by Departmental Ethics Committee, SWPS University. The patients/participants provided their written informed consent to participate in this study.

## Author contributions

The author confirms being the sole contributor of this work and has approved it for publication.

## Conflict of interest

The authors declare that the research was conducted in the absence of any commercial or financial relationships that could be construed as a potential conflict of interest.

## Publisher’s note

All claims expressed in this article are solely those of the authors and do not necessarily represent those of their affiliated organizations, or those of the publisher, the editors and the reviewers. Any product that may be evaluated in this article, or claim that may be made by its manufacturer, is not guaranteed or endorsed by the publisher.

## Appendix

**Table A1:** Descriptive statistics of the variables studied within each identity status.

	Studied variables																			
	Commitment				In-depth exploration				Reconsideration of commitment				BPD symptoms				Depressive symptoms			
	Min	Max	*M*	*SD*	Min	Max	*M*	*SD*	Min	Max	*M*	*SD*	Min	Max	*M*	*SD*	Min	Max	*M*	*SD*
Achievement	3.80	5.00	4.54	0.39	4.20	5.00	4.61	0.28	1.00	2.33	1.12	0.28	49	138	71.24	19.65	0	44	11.01	9.76
Foreclosure	3.00	5.00	3.92	0.41	3.00	4.20	3.87	0.27	1.00	2.33	1.20	0.34	49	154	68.57	15.43	0	48	11.39	9.70
Searching moratorium	1.80	4.00	3.10	0.50	4.00	5.00	4.51	0.23	1.00	3.00	1.62	0.59	52	178	80.88	22.07	0	56	17.62	13.04
Diffusion	1.60	3.80	2.81	0.49	2.60	4.00	3.46	0.33	1.00	2.67	1.56	0.52	51	153	79.88	20.13	0	48	21.06	11.90
Moratorium	1.00	4.00	2.37	0.61	3.00	4.80	3.90	0.43	2.00	4.33	3.22	0.59	51	160	91.41	21.72	1.00	52	24.23	13.10
